# Short-term mindfulness intervention reduces the negative attentional effects associated with heavy media multitasking

**DOI:** 10.1038/srep24542

**Published:** 2016-04-18

**Authors:** Thomas E. Gorman, C. Shawn Green

**Affiliations:** 1 Department of Psychology University of Wisconsin-Madison 1202 W. Johnson St. Madison, WI 53706.

## Abstract

Recent research suggests that frequently switching between various forms of media (i.e. ‘media multitasking’) is associated with diminished attentional abilities, a disconcerting result given the prevalence of media multitasking in today’s society. In the present study, we sought to investigate the extent to which the deficits associated with frequent media multitasking can be temporarily ameliorated via a short-term mindfulness intervention previously shown to produce beneficial effects on the attentional abilities of normally functioning individuals. Consistent with previous work, we found: (1) that heavy media multitaskers showed generally poorer attentional abilities than light media multitaskers and (2) that all participants showed benefits from the short-term mindfulness intervention. Furthermore, we found that the benefits of the short-term mindfulness intervention were not equivalently large across participants. Instead, these benefits were disproportionately large in the heavy media multitaskers. While the positive outcomes were short-lived, this opens the possibility of performing long-term interventions with the goal of realizing lasting gains in this population.

Technological advances over the past decade have made it considerably easier, and in some cases even compulsory, for individuals to engage with multiple streams of media simultaneously - an activity known as ‘media multitasking’. Given that media multitasking entails both constant switches of attention between competing media sources (e.g. switching between email, web browsing, music, etc.) as well as a rather overall diffuse attentional state (e.g. devoting some attention to monitoring for texts even while working on a document), this has led to significant scientific interest in the possible consequences that excessive multitasking may have on attentional abilities.

The research to-date suggests that media multitasking is largely associated with diminished attentional capacities. For instance, Ophir and colleagues^1^, contrasted attentional control in individuals who, as part of their normal life, engaged in large amounts of media multitasking (known as ‘heavy media multitaskers’ or HMMs), with attentional control in individuals who engaged in little media multitasking (known as ‘light media multitaskers’ or LMMs). They found that HMMs performed significantly worse than LMMs on a variety of cognitive tasks, in particular in conditions that required active attentional filtering of distracting stimuli. Since this original report, many follow-up investigations have found similar associations between large amounts of media multitasking and diminished attentional abilities suggesting that the base effect is reliable^2^,^3^,^4^,^5^,^6^ (although see^7^,^8^).

While media multitasking is associated with impaired attentional abilities, a growing body of research suggests that the same set of skills that may be harmed by media multitasking can be enhanced in normal functioning individuals. Activities shown to be beneficial for cognitive skills include playing action video games^9^, music training^10^, interactions with nature^11^, and mindfulness meditation^12^. Mindfulness is of particular interest, as the mechanism through which mindfulness is thought to improve attention (including narrowing focus and reducing mind-wandering) is the opposite of the broad, poorly filtered attention thought to underlie the negative effects seen in HMM. Mindfulness may also be a particularly useful tool in that it has been shown to have benefits even in short bouts. For example, Mrazek and colleagues^13^ found that just 8 minutes of breath-focused meditation led to improved performance on a sustained attention task as compared to both reading and passive relaxation control groups. Similarly, Sormaz and colleagues^14^ showed that thirty minutes of a guided, breathing meditation led to clear enhancements in distractor filtering.

The main question of the present investigation is whether a short-term mindfulness meditation intervention can temporarily ameliorate the deficits associated with heavy media multitasking. To address this question, both heavy and light media multitaskers were asked to perform a series of attentional tasks either directly after a bout of mindfulness meditation or after web browsing for the same length of time (in a within-participants design, i.e. - participants experienced both types of short-term intervention on different days). We had three primary predictions: 1) that, in a manner consistent with previous results, HMM participants would perform overall worse on the attentional tasks than LMM participants; 2) that, again in a manner consistent with previous results, both LMM and HMM participants would perform better on the attentional tasks after completing the short-term mindfulness intervention than after completing the control intervention; and 3) that the HMM participants would perform disproportionately better in the context of the short-term mindfulness intervention as compared to the LMM participants, due to the fact that HMM are likely to have a less focused default attentional state and thus should reap greater benefits from an intervention that encourages a more focused attentional state.

## Methods

### Participants

The Media Multitasking Index (MMI)^1^ was administered online to 1,683 undergraduate students enrolled in Introductory Psychology at the University of Wisconsin-Madison during the Fall 2014 and Spring 2015 semesters alongside a number of other surveys that are not relevant to the current manuscript. The mean MMI score from our sample was 3.72 with a standard deviation of 1.70. These values are similar to those that have been found by other groups that have utilized college samples^1^,^15^,^16^. From this sample of 1,683 individuals we covertly recruited 48 participants (i.e. participants were recruited without knowledge that they were selected based upon their media usage). This number of participants was within our a priori goal range of 20–25 participants per group, which was determined based upon previous work in the domain (our recruitment ceased at the end of the Spring 2015 semester). Our criteria for recruitment was that an individual must have had an MMI score either greater than 1 SD above the mean of the sample (e.g. 5.42 for individuals that would be classified as heavy media multitaskers - HMMs) or less than 1 SD below the mean of the sample (e.g. 2.02 for individuals that would be classified as Light Media Multitaskers - LMMs). Six participants (2 LMM, 4 HMM) were excluded from further data analysis for either abnormally poor performance (e.g. having scores on multiple measures more than 3 SD below the mean) or for failing to follow task instructions (e.g. falling asleep during a task). Of the 42 participants that remained and that make up our final sample, 22 were classified as HMMs (mean MMI = 7.28, 17 Females, 5 Males) and 20 were classified as LMMs (mean MMI = 1.02, 12 Females, 8 Males). All participants provided informed, written consent and received either $10/hr or course credit for their participation. This study was approved by the Education and Social/Behavioral Science Institutional Review Board (IRB) at the University of Wisconsin-Madison. All methods were carried out in accordance with the approved guidelines.

### Apparatus

All cognitive performance measures were completed on a Dell OptiPlex computer running MATLAB Programming Environment Version 2014a and the Psychophysical Toolbox^17^,^18^. Visual stimuli were presented on an LCD display at a viewing distance of approximately 59 cm.

### Overall Design

The experiment consisted of two sessions, each of which took approximately 110 minutes in total and were completed on separate days no more than 48 hours apart. In each session the participant underwent a battery of tasks designed to assess attentional control and working memory (see task battery below). The tasks were interspersed between 10-minute bouts of one of two types of short-term intervention that differed by session. In one session the behavioral tasks were interspersed between bouts of an intervention designed to induce a mindful state (breath counting, see below). In the other session, the tasks were interspersed between bouts of a control intervention designed to mimic an activity in which most college individuals often engage (web browsing – see below). Half of the participants engaged in the breath counting intervention on the first day, and the web browsing intervention on the second day; the other half of participants completed the breath counting and web browsing in the opposite order.

**Figure 1:**
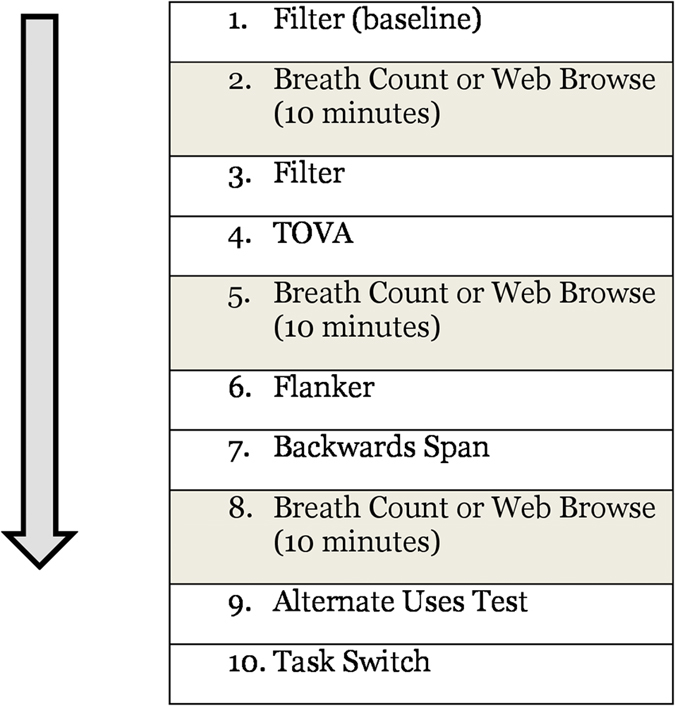
General Task Design. In each session, cognitive tasks (white boxes) were interspersed with 10-minute bouts of a short-term intervention predicted to improve attentional performance (i.e. breath counting) or a control intervention (i.e. web browsing – gray boxes).

As shown in Fig. 1, participants first completed the filter task as a baseline measure. They then engaged in an initial 10-minute bout of their respective short-term intervention (i.e., either breath counting or web browsing). Next, they completed the filter task again, followed by the impulsivity task. Participants then engaged in a second 10-minute bout of their respective short-term intervention. This was followed by completion of the flanker task and the backwards digit span. Participants then completed a 10-minute bout of their respective intervention for a third and final time, after which they immediately filled out the flow state scale (to assess their feelings about the intervention itself). Finally, participants completed the alternate uses test and the task switching task. The second session was identical to the first in all regards except for: (1) the short-term intervention (i.e., those who underwent breath counting in session 1, switched to web browsing in session 2 and vice versa) and (2) at the conclusion of session 2 participants filled out the Mindful Attention Awareness Scale^19^ and a survey of their current and past video game use^20^.

### Short-term interventions

#### Breath Counting

The breath counting task was identical to that utilized by Levinson and colleagues^21^ with the exception of the duration. In the task participants were instructed to count their breaths while simultaneously pressing the down arrow key on the keyboard with each exhale. On every 9th breath, participants were asked to press a different key (the right arrow key) and then start their count over from zero. While counting their breaths, participants viewed slowly moving, animated natural stimuli. Visual feedback was provided if they made a mistake in their count (i.e. if they pressed the right arrow key after a breath other than the 9^th^ breath). Although true breathing rate was not explicitly tracked, previous research has demonstrated a strong correspondence between button presses and actual breathing rates in this task^21^. Participants underwent three 10-minute bouts of this exercise within a single session interspersed between various behavioral tasks (see Fig. 1).

#### Web browsing

The web browsing activity was designed to act as a control condition against which the effect of the breath counting could be compared. It was thus important to us to utilize a task that would mimic a real-life activity in which participants may engage. Thus, for the web browsing intervention, participants were allowed to alternate between three different websites (Wikipedia, The Huffington Post, Buzzfeed), and were told they could browse however they liked, so long as they remained engaged on one of those three sites at all times. As with the breath counting task, participants underwent three 10-minute bouts of this short-term intervention within a session interspersed between various behavioral tasks (see Fig. 1). It is important to note that this form of web browsing is not an example of media multitasking, as participants were not allowed to engage with other forms of media simultaneously (i.e. they were not allowed to talk on their phones, text, email, or listen to music during the web-browsing portions). Thus, this web-browsing intervention was meant to be equally applicable to both the HMM and LMM participants – as nearly all college students spend a significant amount of time engaged in web-browsing.

### Task Battery

#### Attention Tasks

##### Filter Task

The filter task was modeled after that utilized by Ophir^1^, but with a slightly reduced stimulus set. In this task participants were briefly presented (100 ms) with an array of two red target rectangles and either two or ten blue distractor rectangles in each trial. The rectangles could be oriented either vertically, horizontally, or along either of the two diagonals. Then, after a 900 ms blank interval, participants were shown a second display. On half of the trials, this second display was identical to the first display. On the other half of the trials, the orientation of one of the red target rectangles was changed in the second display (the blue distractor rectangles never changed). Participants were asked to indicate whether the red rectangles were the same or different from the original array. Participants were asked to respond as accurately as possible without time pressure and thus the sole dependent measure for this task was sensitivity (d’).

##### Impulsivity

The impulsivity task was modeled after a portion of the Test of Variables of Attention^22^. On each trial participants were presented with a square that could appear either on the top half or the bottom half of the screen. If the square appeared in the top half of the screen (80% of trials), participants were asked to press the up arrow key as quickly as possible (i.e., a “go” trial). If the square appeared in the bottom half of the screen (20% of trials), they were asked to make no response (“nogo” trial). The measured variables in this task thus included: (1) reaction time (RT) on correct trials and (2) the number of incorrect responses – which includes both “go” responses on “nogo” trials and any responses made before the square appeared.

#### Flanker Task

The flanker task was modeled after the Eriksen flanker task^23^. On each trial participants were shown 5 shapes presented along the horizontal meridian. The center shape was always an arrow that faced either left or right. The participants’ task was to indicate this arrow’s direction as quickly and accurately as possible by pressing the corresponding arrow key on the keyboard. The flanking shapes could be response compatible (i.e. all pointing in the same direction as the center arrow), response incompatible (i.e. all pointing in the opposite direction as the center arrow) or response neutral (i.e. consisting of rectangle shapes rather than arrows). The measured variables in this task thus included: (1) RT and (2) accuracy.

##### Task-Switch

The task switch task was modeled after Rogers and Monsell^24^. On each trial participants were shown a number (from 1–9; excluding 5) within a colored (blue or yellow) square. On trials where the square was colored yellow, participants were asked to categorize the number as either even or odd by pressing either the ‘>’ or the ‘ ?’ key on the keyboard respectively. On trials where the square was blue, participants were asked to categorize the number as either high (greater than five), or low (less than five) by pressing the ‘Z’ or the ‘X’ key on the keyboard respectively. The task switched predictably every 3 trials. The measured variables in this task thus included: (1) RT and (2) accuracy.

#### Other Tasks

Our hypothesis was that the breath counting task would primarily affect attentional tasks. To assess whether this short-term intervention altered more general cognition as well, a single working memory task and a single cognitive flexibility task were also included in the battery.

##### Backwards Digit Span

In this task participants were presented with a stream of digits (no repeats) of five possible lengths (3, 4, 5, 7, 9). The digits appeared in the center of the screen one at a time at a rate of 1/sec. Once the final number was presented, participants were instructed to write down the stream of numbers in the reverse order of which they appeared. The measured variable in this task was thus only accuracy.

##### Alternate Uses

This task was modeled after Colzato^25^. On each trial participants were given the label of a common object (i.e. brick, towel, newspaper), and were asked to list as many distinct uses for that item as they could think of, in a 3 minute timespan. The responses were coded into three different categories: 1) Fluency: the number of individual responses. 2) Flexibility: the number of distinct categories used in the responses (i.e. using a brick to build a house and to build a garage, fall under the same category). 3) Elaboration: the amount of detail included in each response.

## Results

### Data Processing

The analyses for the three tasks that included an RT component (i.e. flanker, impulsivity, task-switching) were similar to those used by Cardoso and colleagues^5^. For each participant/task/condition, we first log transformed the RTs and removed any aberrant outlying RTs (2.5 SD or greater beyond the mean). We then returned the RTs to normal space and computed an inverse efficiency measure by dividing their response speed by their accuracy. For the filter task, performance was assessed in terms of sensitivity (d’) separate by distractor condition. For the backwards span task, we found that accuracy between set sizes 5 and 9 was well fit by a linear regression (i.e., over this range performance decreased linearly without being at either ceiling or floor). Thus for each participant we found the best fitting line and calculated an 80% threshold. For the alternate uses task we combined fluency, flexibility and elaboration scores into a single score.

Given the complexity of the design, we first examined performance between sessions to determine if there was a day effect, and found that there was no significant effect of day or interactions between group and day or group, day, and condition. We then examined performance in the three tasks that had multiple sub-conditions (e.g. filter: 2 distractors/10 distractors; flanker: congruent/incongruent/neutral; task-switching: repeat/switch) to determine if it was appropriate to reduce performance down to a single metric. In each case ANOVAs were run with MM group (HMM/LMM) and the respective sub-conditions in the given task as factors. No significant interactions between group and sub-condition were found (see Supplemental Materials for additional detail). This is consistent with our previous work, which has shown globally poor performance of HMM individuals as compared to LMM individuals, rather than disproportionately poor performance in HMM individuals only in certain sub-conditions. Thus, for each of these tasks we collapsed across sub-condition for our analyses of interest.

**Figure 2:**
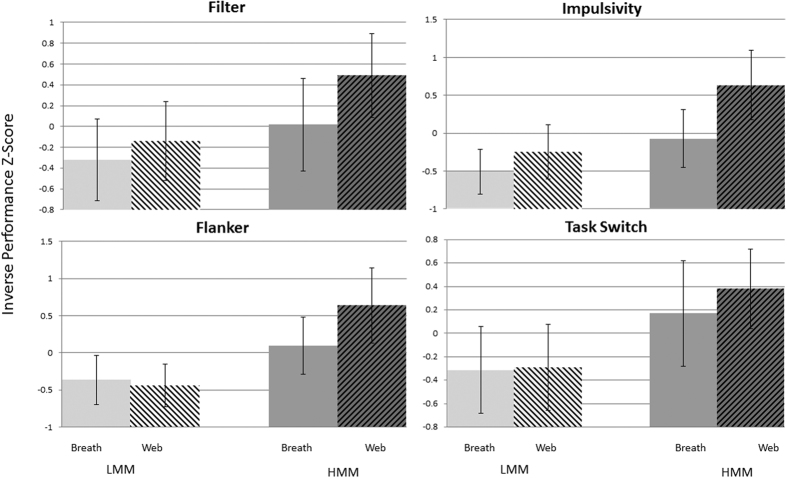
Results – Attentional Tasks (y-axis is inverse performance z-scores, thus lower/more negative scores indicate better performance). Across all four tasks (filter, impulsivity, flanker, and task switch) three main trends are present. 1) LMM individuals generally outperform HMM individuals on all tasks. 2) Overall participants perform the tasks better after breath counting than after web browsing. 3) The beneficial effect of breath counting is disproportionately large in HMM individuals as compared to LMM individuals. Error bars indicate 95% confidence intervals.

To test our primary hypotheses – (1) that HMM individuals would perform overall more poorly than LMM individuals in measures of attention; (2) that all individuals would perform better on measures of attention during the session with the breath counting task than during the session with the web browsing activity; and (3) that HMM individuals would show a disproportionate benefit from the breath counting task – we first transformed the performance measures to z-scores (to ensure that performance measures on all four attention tasks were on the same scale) and then conducted a MANOVA with short-term intervention condition (Breath Counting vs. Web browsing) as a within-subjects factor and MM group (HMM vs. LMM) as a between-subjects factor. Consistent with our first hypothesis, we found a significant main effect of MM group (F(1,40) = 11.997, p = 0.001, 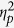 = 0.231), with HMM individuals showing overall poorer performance (i.e. higher z-values) than LMM individuals across the four attentional tasks. Consistent with our second hypothesis, we also found a significant effect of short-term intervention condition (F(1,40) = 9.87, p = 0.003, 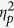 = 0.198), with individuals performing overall better on the attentional measures in the context of the breath counting task. And finally, consistent with our third hypothesis, we found a significant interaction between MM group and short-term intervention condition (F(1,40) = 4.384, p = 0.043, 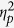 = 0.099) with the HMM individuals showing a disproportionate benefit across attentional tasks in the context of the breath counting task as compared to the LMM individuals (see Fig. 2). As further predicted, these effects were only observed for attentional measures as no main effects nor interaction was observed for the working memory or cognitive flexibility measures (see Supplemental Materials for additional detail).

## Discussion

Here we investigated the effect of a short-term mindfulness intervention on the attentional skills of individuals who were either light or heavy media multitaskers. Previous results have shown that individuals who engage in heavy media multitasking show diminished performance on attentional measures as compared with individuals who rarely engage in media multitasking^1^,^2^,^3^,^4^,^5^,^6^. Research has also shown that undergoing mindfulness interventions can produce enhancements in attentional measures^12^,^13^,^14^. Consistent with these distinct literatures, both base effects were replicated in our data. Of particular note is the fact that, mirroring several recent results, HMM individuals were found to be generally poor across the board on measures of attention, rather than being deficient solely in certain task conditions^1^,^5^. Our study also found no relationship between media multitasking and backwards span performance, which echoes the lack of an effect seen in n-back performance in the seminal study by Ophir and colleagues^1^. However, note that a recent study by Uncapher and colleagues^6^ found that HMM participants performed worse on multiple working memory measures involving a task similar to the filter task described in the present manuscript, but construed to load on working memory as opposed to selective attention. By this construal, our results may also be in line with theirs, suggesting that heavy media multitasking is associated with decrements in certain working memory tasks.

Of particular interest for this report though was the confluence of the two effects – media multitasking and mindfulness mediation. The beneficial effects of mindfulness interventions on attention (e.g. increased focus, decreased mind wandering) are believed to be roughly opposite to the negative effects associated with heavy media multitasking (e.g. deficits in attentional control, overly diffuse attention). This leads directly to the prediction that the benefits of a short-term mindfulness intervention should be disproportionately large in heavy media multitaskers (whose performance is limited by improper/diffuse attentional focus) as compared to light media multitaskers. And consistent with these a priori predictions, HMM participants showed disproportionate improvements on the attentional measures after the mindfulness exercise as compared to the LMM participants. Although in our case the intervention was short, and thus the beneficial effects were only transient (i.e., participants who engaged in the breath counting game on their first day of the experiment did not show any persisting improvements in their performance on the second day), this data suggests that the attentional state of HMMs can be modified via experience. Because in our design the mindful state was constantly refreshed (i.e., the participants engaged in the breath counting exercise every 10–15 minutes) we cannot say exactly how long the effect persisted, it is clear that a long-term intervention study is warranted in order to determine the dose-response curve associated with true mindfulness training and whether it is possible to produce more lasting improvements in HMM individuals.

Such a long-term intervention study could also make use of additional control experiences. Indeed, given our current design we cannot rule out the possibility that rather than the breath counting condition being disproportionately beneficial for the HMM participants, it could instead be the case that the web browsing condition was disproportionately harmful to the HMM participants. We chose web browsing as our control condition not because we consider it to be a true baseline (it is unclear if there even can be a true baseline for studies examining transient effects on attention), but because we believe that unlike breath counting, web browsing is a type of activity that all of our subjects are likely to engage in during their day to day lives. Additional control conditions though would allow one to better determine whether mindfulness disproportionately *aids* performance, or if instead web-browsing disproportionately *diminishes* performance in HMMs.

In all, the current results should strongly inform future work in a field that continues to grow in importance as the prevalence of media multitasking rises. Given that the types of capabilities harmed by media multitasking are strongly associated with real-world outcomes (i.e. academic performance, employment, general well-being), developing effective methods to ensure that individuals who would otherwise have diminished cognitive faculties are capable of functioning at optimal levels is of critical importance.

## Additional Information

**How to cite this article**: Gorman, T. E. and Green, C. S. Short-term mindfulness intervention reduces the negative attentional effects associated with heavy media multitasking. *Sci. Rep.* **6**, 24542; doi: 10.1038/srep24542 (2016).

## Supplementary Material

Supplementary Information
