# Supplementary material for: Short-term mindfulness intervention reduces the negative attentional effects associated with heavy media multitasking
Source: Sci Rep. 2016 Apr 18;6:24542. doi: 10.1038/srep24542 (PMC4834474; doi:10.1038/srep24542)
Supplement: Supplementary Information [file srep24542-s1.doc]

**Short-term mindfulness intervention reduces the negative attentional effects associated with heavy media-multitasking**

Thomas E. Gorman^1^ & C. Shawn Green^1^

1 – Department of Psychology

University of Wisconsin-Madison

1202 W. Johnson St.

Madison, WI 53706

Corresponding Author: Thomas E. Gorman – tegorman@wisc.edu

**Supplementary Analyses**

Given the overall complexity of the design, we first examined whether it was appropriate to reduce performance in each of the tasks that included multiple sub-conditions (e.g. filter: 2/10 distractors; flanker: compatible/incompatible/neutral flankers; task-switch: switch/non-switch trials) by performing, for each respective task, an ANOVA that included the given sub-conditions along with MM group (HMM/LMM) and intervention condition (breath counting/web browsing). In particular we were interested in whether, for any task, the given sub-conditions interacted with either MM group or intervention condition (which would suggest that collapsing across task sub-condition would be inappropriate). In no cases though did we observe a significant interaction between task sub-condition and either MM group or intervention condition (or a three-way interaction between task sub-condition, MM group, and intervention condition) – see below.

*Filter*

An ANOVA on z-scored d’ values with within-subjects factors: distractor set size (2 distractors/10 distractors), and intervention condition (breath count/web browsing) and between subject factor MM group (HMM/LMM) revealed no significant interaction between distractor set size and MM group (F,1,40)= 1.191, p = .282), distractor set size and intervention condition (F(1,40) = .126, p = .724), or the three way interaction between distractor set size, MM group, and intervention condition F(1,40) = .126, p = .724).

*Flanker*

An ANOVA on z-scored inverse efficiency scores with within-subjects factors: distractor compatibility (compatible/incompatible/neutral), and intervention condition (breath count/web browsing) and between subject factor MM group (HMM/LMM) revealed no significant interaction between distractor compatibility and MM group (F(2,39) = .283, p = .755) or distractor compatibility and intervention condition (F(2,39) = 2.464, p = .098) or the three way interaction between distractor set size, MM group, and intervention condition (F(2,39) = .155, p = .857).

*Task Switch*

An ANOVA on z-scored inverse efficiency scores with within-subjects factors: switch condition (switch/no switch), and intervention condition (breath count/web browsing) and between subject factor MM group (HMM/LMM) revealed no significant interaction between switch condition and MM group F(1,40) = .160, p = .691), switch condition and intervention condition (F(1,40) = .408, p = .527), or the three way interaction between distractor set size, MM group, and intervention condition (F(1,40) = .511, p = .479)

**Untransformed Data**

Presented in tables below are the group and intervention data for each task included in the main analysis, as well as the backwards span task.

Filter

|  | **Breath Counting** |  | **Web Browsing** |  |
| --- | --- | --- | --- | --- |
|  | Sensitivity 2 Distractors | Sensitivity 10 Distractors | Sensitivity 2 Distractors | Sensitivity 10 Distractors |
| **LMM** | .528(.169) | .367(.129) | .489(.164) | .357(.152) |
| **HMM** | .441(.126) | .338(.170) | .399(.198) | .296(.129) |

*Impulsivity*

|  | **Breath Counting** |  | **Web Browsing** |  |
| --- | --- | --- | --- | --- |
|  | Median RT | % trials correct | Median RT | % trials correct |
| **LMM** | 0.295(.030) | 0.974(.024) | 0.305(.0409) | 0.968(.021) |
| **HMM** | 0.303(.038) | 0.951(.035) | 0.327(.039) | 0.940(.063) |

*Flanker*

|  | **Breath Counting** |  |  |  |  |  |
| --- | --- | --- | --- | --- | --- | --- |
|  | Cmpt RT | Cmpt % correct | Incmpt RT | Incmpt % correct | Neutral RT | Neutral % correct |
| **LMM** | 0.391(.048) | 0.997(.012) | 0.435(.051) | 0.941(.038) | 0.386(.050) | 0.993(.012) |
| **HMM** | 0.421(.071) | 0.997(.008) | 0.470(.067) | 0.949(.060) | 0.413(.054) | 0.989(.020) |

|  | **Web Browsing** |  |  |  |  |  |
| --- | --- | --- | --- | --- | --- | --- |
|  | Compt RT | Cmpt % correct | Incmpt RT | Incmpt % correct | Neutral RT | Neutral % correct |
| **LMM** | 0.381(.036) | 0.993(.017) | 0.426(.044) | 0.954(.034) | 0.386(.041) | 0.991(.013) |
| **HMM** | 0.445(.069) | 0.998(.006) | 0.497(.076) | 0.954(.044) | 0.446(.077) | 0.993(.012) |

*Task Switch*

|  | **Breath Counting** |  |  |  | **Web Browsing** |  |  |  |
| --- | --- | --- | --- | --- | --- | --- | --- | --- |
|  | No Switch RT | No Switch % correct | Switch RT | Switch % Correct | No Switch RT | No Switch % correct | Switch RT | Switch % Correct |
| **LMM** | 0.600(.070) | 0.890(.056) | 0.957(.217) | 0.914(.057) | 0.601(.082) | 0.886(.058) | 0.961(.297) | 0.903(.057) |
| **HMM** | 0.678(.150) | .874(.057) | 1.02(.250) | .883(.068) | .679(.118) | .852(.063) | 1.06(.190) | .868(.064) |

*Backwards Span*

|  | **Breath Counting** |  |  |  |  | **Web Browsing** |  |  |  |  |
| --- | --- | --- | --- | --- | --- | --- | --- | --- | --- | --- |
|  | 3 span | 4 span | 5 span | 7 span | 9 span | 3 span | 4 span | 5 span | 7 span | 9 span |
| **LMM** | .995(.018) | .966(.052) | .954(.057) | .783(.143) | .593(.169) | .987(.030) | .976(.060) | .950(.071) | .743(.164) | .563(.136) |
| **HMM** | .970(.060) | .938(.105) | .940(.099) | .741(.149) | .554(.140) | .981(.061) | .968(.054) | .904(.106) | .744(.154) | .591(.173) |
